# Acute Upper Extremity Ischaemia as a Result of Muscular Hypertrophy: The Popeye Syndrome

**DOI:** 10.7759/cureus.48556

**Published:** 2023-11-09

**Authors:** Konstantinos G Seretis, Sofia Tzamtzidou, Theofanis Papas

**Affiliations:** 1 Department of Vascular Surgery, Korgialenio-Benakio Hellenic Red Cross Hospital, Athens, GRC

**Keywords:** lacertus fibrosus, bicipital aponeurosis, muscular hypertrophy, acute upper extremity ischaemia, arterial entrapment syndrome

## Abstract

Arterial entrapment syndrome (AES) at the elbow level is very rare. In cases of acute upper extremity ischaemia presenting in middle-aged patients with evident muscular hypertrophy, AES should always be included in the differential diagnosis. A thorough clinical examination should always follow, particularly when symptoms appear after reported strenuous upper extremity activity, and emergent surgical decompression is mandatory to avoid thrombotic complications in the affected arm in the future.

## Introduction

Acute upper extremity ischaemia caused by brachial artery entrapment at the elbow level is very rare and has always been described in individuals doing physically demanding jobs or athletes with evident muscular hypertrophy [1, 2]. Compression of the brachial artery in the elbow region is caused by bicipital aponeurosis (BA), also known as lacertus fibrosus, which represents a thickening of the brachial fascia that joins the biceps brachii muscle to the ulna. Bicipital aponeurosis serves to protect the underlying neurovascular bundle, and anatomical variations in origin and size have been described [3, 4]. According to some authors, a thickened BA can compress the underlying structures, median nerve, and brachial artery, resulting in symptoms of mixed aetiology: motor and sensory symptoms due to compression of the median nerve [5-7], and ischemic symptoms due to compression of the brachial artery. We present a case of a patient who presented with acute-onset left upper extremity ischaemia due to compression of the brachial artery by BA.

## Case presentation

**Figure 1:**
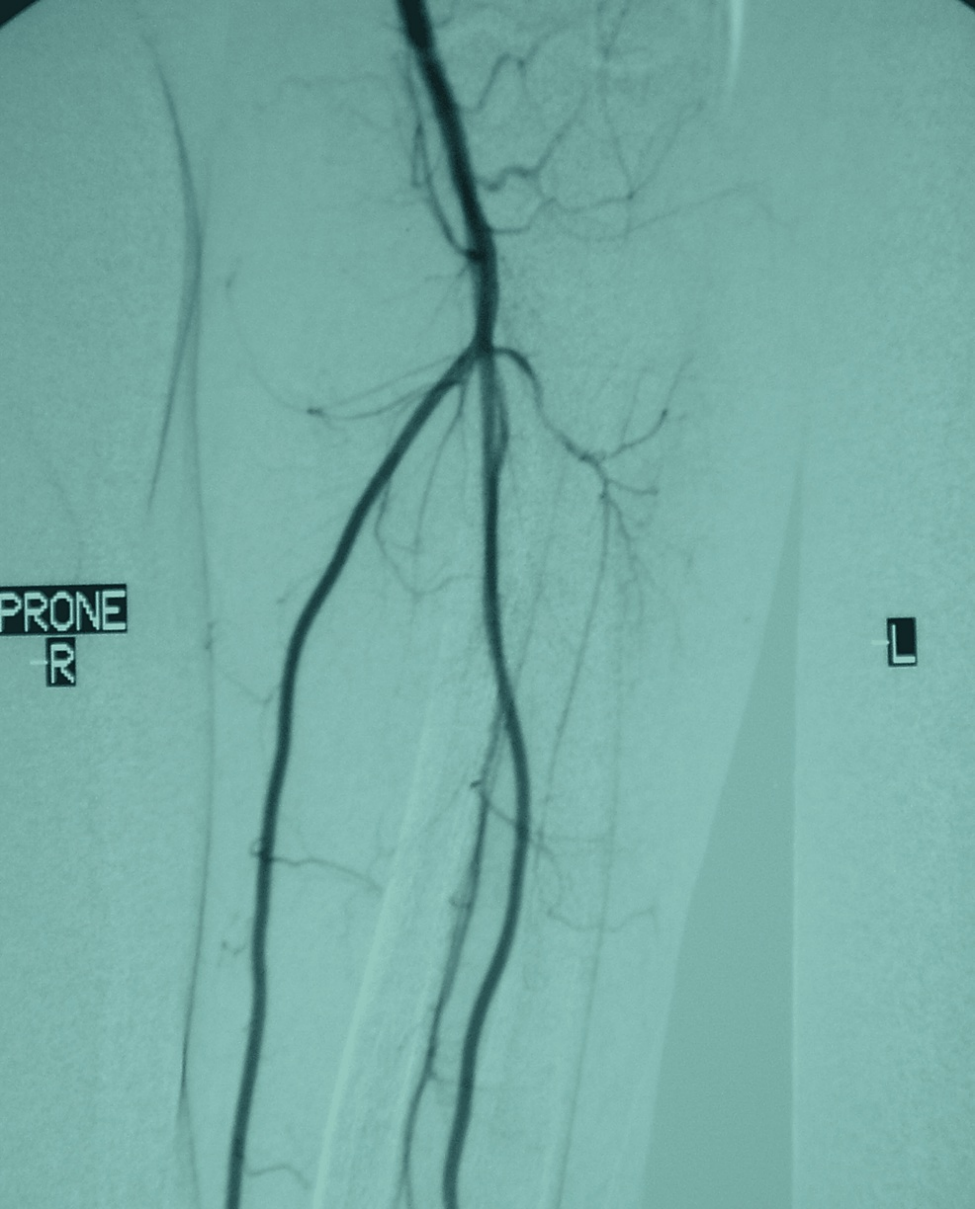
Dynamic angiography of the left upper extremity upon admission with the arm in anatomical position and muscles at rest

**Figure 2:**
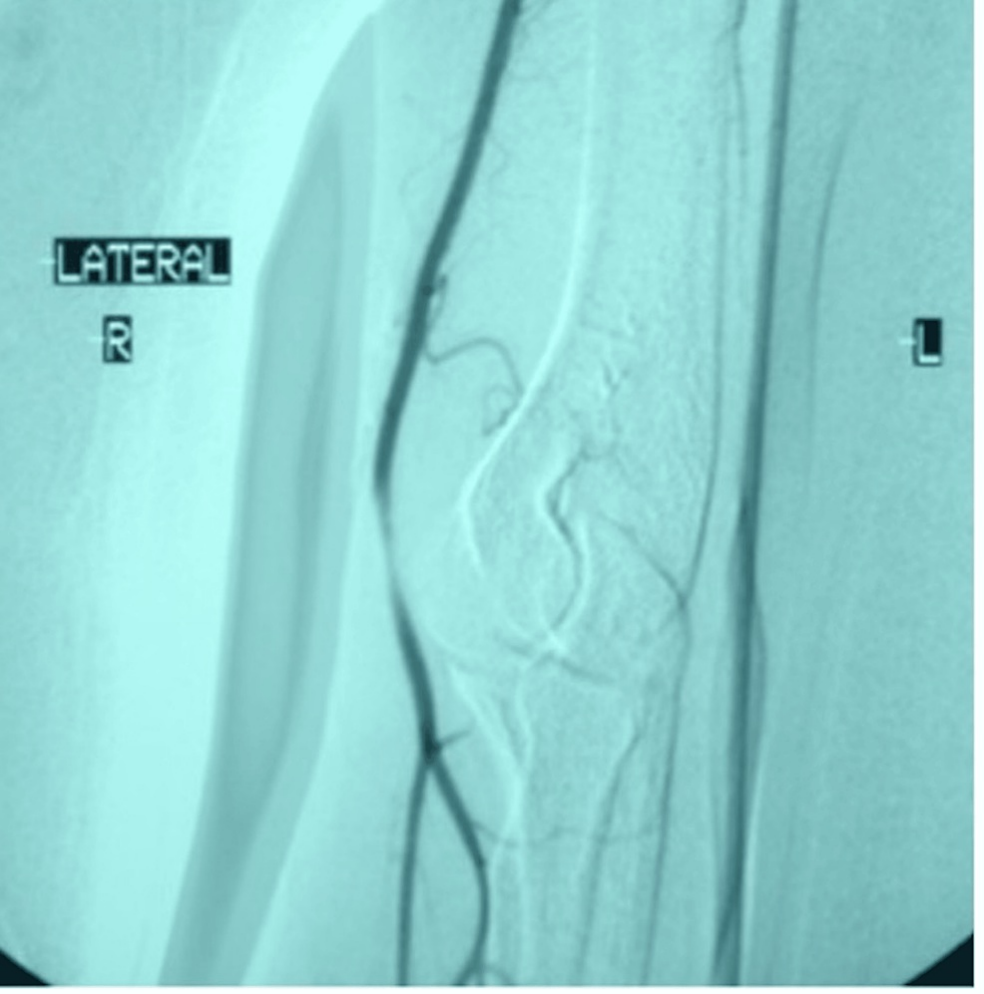
Dynamic angiography of the upper extremity upon admission with the arm in pronation position and muscles at rest

**Figure 3:**
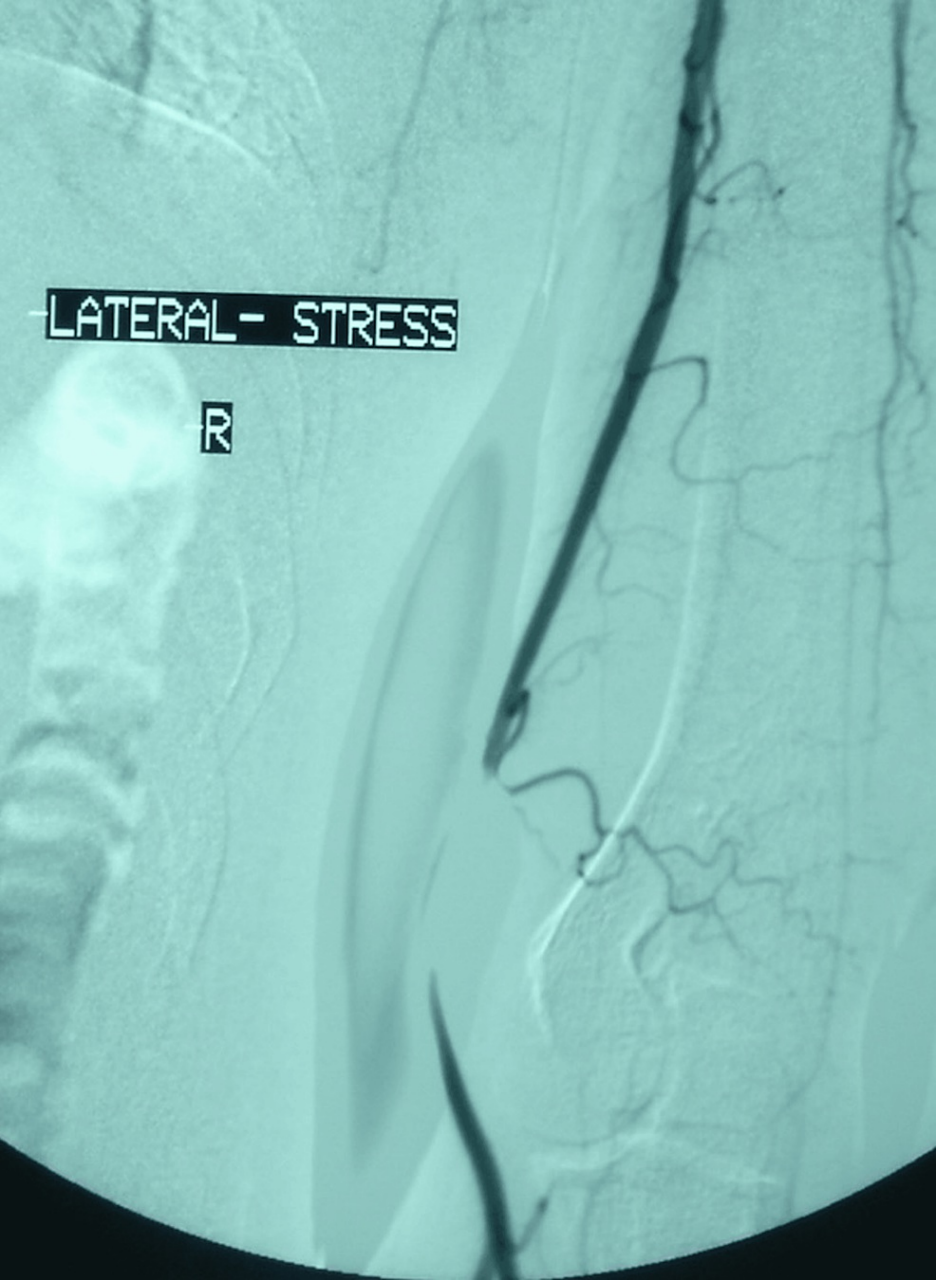
Dynamic angiography of the upper extremity upon admission with the arm in pronation position and muscles stressed

We report a case of entrapment syndrome of the brachial artery that was successfully treated in our hospital. Our patient, a 39-year-old construction worker, presented with numbness and coldness in his left hand after vigorous physical activity at work. Because of the nature of his work, working for hours with his hands patient had developed a profound muscular hypertrophy in both arms. During the clinical examination, his left arm was pale and cold, and his fingers were numb. Palpable pulses were present in the radial and ulnar arteries, respectively, but they disappeared after pronation and contraction of the biceps brachii muscle. The patient was transferred to the angiographic suite, and selective angiography of the arteries of his left upper extremity was done. The angiography (Figures 1-3) revealed a smooth compression in the distal brachial artery when the arm was in pronation, while the compression was more intense and led to near-total occlusion of the artery when the muscles of the arm were contracted.

This intermittent compression was caused exclusively by muscular hypertrophy, and the diagnosis of entrapment syndrome of the brachial artery was set.

The patient was transferred to the operating room for open repair, and under general endotracheal anaesthesia, decompression of the brachial artery was achieved. More specifically, the left brachial artery was dissected from the level of the mid-arm to the level of its bifurcation into radial and ulnar, and the ulnar aponeurosis of the biceps brachii muscle, also known as bicipital aponeurosis or lacertus fibrosus, was transected. Immediate post-operative recovery of pulses in the radial and ulnar arteries in all positions of the upper extremity was evident, and after cutting the lacertus fibrosus, the brachial artery was totally liberalised and symptoms completely disappeared. The hospital stay was three days, and the patient remained free of symptoms with palpable pulses in the radial and ulnar arteries during the course of his post-operative follow-up.

## Discussion

Lacertus fibrosus has been associated with compression at the elbow level, and numerous reports of neural compartment syndrome have been described [5-7]. Entrapment of the brachial artery was first described in a young male patient in 1977 [8], and only a few cases have been reported ever since [9,10]. Clinical presentation includes the acute onset of ischemic symptoms that may be accompanied by symptoms of the surrounding peripheral nerves. The condition causing arterial entrapment of the arteries at the elbow level is associated with physically demanding jobs or athletes, and muscular hypertrophy of the upper extremities is always evident. In most cases, the patient experiences atypical intermittent upper extremity claudication with relief of the symptoms at rest, and a high level of clinical suspicion is required for the diagnosis to be set. It is important to identify the level of compression and differentiate brachial artery entrapment at the elbow level from compressive syndromes that occur more proximally in the upper extremity, i.e., arterial thoracic outlet syndrome (TOS) [11]. A differential diagnosis is made after careful evaluation of the patient’s history and a thorough physical examination, and multiple diagnostic modalities may be used to recognise the site of compression. These include magnetic resonance angiography or digital subtraction angiography combined with provocative maneuvers of the upper extremity. Examination during arm abduction, at rest, in pronation, and in pronation along with muscle contraction provides images characteristic of each condition. In cases of intermittent upper extremity claudication with relief of the symptoms at rest, non-operative management may be considered. This includes rest, activity modification, and the use of anti-inflammatory drugs to reduce the oedema of the thickened BA, and the majority of patients will benefit from this conservative treatment. Surgical management is considered after failure of non-operative management and as the first-line option in cases of acute arterial ischemia. Acute arterial ischemia is an emergent condition that requires immediate repair because of the associated risk of arterial thrombosis. Surgical repair is simple and involves decompression of arterial entrapment that is achieved by cutting lacertus fibrosus, which results in total relief of the related complaints and symptoms.

## Conclusions

Patients with acute intermittent upper extremity ischaemia due to brachial artery compression by the lacertus fibrosus are a rare entity, and lacertus fibrosus has been related to intermittent arterial compression in only a few cases. Muscular hypertrophy related to excessive muscle use is always evident, and the lacertus fibrosus is responsible for the development of arterial compression, resulting in compartment syndrome in the affected arm. Entrapment of the brachial artery requires a high degree of clinical suspicion and should be included in the differential diagnosis of acute upper extremity ischaemia presenting in patients with evident muscular hypertrophy.
